# A Rare First Presentation of Unilateral Fibromuscular Dysplasia Resulting in Renal Infarct

**DOI:** 10.7759/cureus.81424

**Published:** 2025-03-29

**Authors:** Kevin Liu, Beata Reilly, Ashley Barnhill, Salman Shah, Anantha Ramanathan

**Affiliations:** 1 Department of Surgery, Nassau University Medical Center, East Meadow, USA; 2 Department of Radiology, Stony Brook University, Stony Brook, USA; 3 Department of Radiology, Nassau University Medical Center, East Meadow, USA

**Keywords:** african american female, balloon angioplasty, fibromuscular dysplasia, older presentation, renal infarct

## Abstract

A rare first presentation of unilateral fibromuscular dysplasia (FMD) in an African American patient in her 60s, with renal infarct, is described, along with imaging. The patient presented with acute-onset right-sided abdominal pain and severe hypertension not responding to multiple antihypertensives. She was found to have renal infarcts on the right side. Further imaging with computed tomography angiography (CTA) and duplex scan were suspicious for FMD on the right side. The diagnosis was confirmed by selective renal digital subtraction angiography, and the patient underwent a successful balloon angioplasty of the right renal artery with resolution of her symptoms and hypertension. The presentation is unusual because of her age and ethnicity. The case is discussed along with differential diagnoses. This case also illustrates that socioeconomic disparities can affect early diagnosis, and eventually the outcome, in healthcare.

## Introduction

Fibromuscular dysplasia (FMD) is a collection of rare non-inflammatory and non-atherosclerotic vascular diseases that predominantly affect young and middle-aged Caucasian women aged 15-50 years [1,2]. Approximately 75% of cases affect the renal arteries and present with secondary, treatment-resistant hypertension, with 35%-50% of those cases involving the bilateral renal arteries [1,3]. Only about 10% of renovascular hypertension is believed to be accounted for by FMD [3]. The etiology of this disease is not well understood, but a variety of factors, including genetic, hormonal, and mechanical, have been suggested. It is most common among first-degree relatives [2,4]. Cigarette smoking and a history of hypertension are also associated with an increased risk of this condition [5]. A history of ever smoking increased the risk by 4.1 times, and there was a probable dose-dependent relationship [5]. Similarly, a history of longer than five years of hypertension was also associated with FMD [5].

The mainstay of diagnosis is imaging [2]. The commonly utilized imaging modalities in the diagnosis of fibromuscular dysplasia (FMD) include non-invasive vascular imaging techniques such as duplex ultrasound, computed tomography angiography (CTA), and magnetic resonance angiography (MRA). Digital subtraction angiography remains the gold standard for diagnosis. Doppler ultrasound remains the first-line screening test for FMD. Computed tomography angiography (CTA) is one of the best tools available to diagnose FMD. Magnetic resonance angiography (MRA) has comparable sensitivity and specificity to that of CTA but without the risk of radiation exposure and contrast-induced nephropathy. Conventional angiography remains the gold standard in diagnosing FMD and can also measure the pressure gradient across the stenotic lesions.

The main differential diagnosis is atherosclerotic disease of the renal arteries. The latter usually involves the origins of the renal arteries, while FMD involves the mid- and distal segments [4]. Other syndromes that may be associated with FMD include Ehlers-Danlos syndromes [6], Alport syndrome and pheochromocytoma [4], Marfan syndrome [7], and Takayasu's arteritis [8]. Some of the above are only based on case reports, and many pertain to other vascular territories [4-8].

Generally, acute phase reactants are normal in FMD unless there is associated infarction, whereas these are elevated in vasculitides [4].

Historically, the treatment for this condition was surgical revascularization, but with the advances in endovascular therapy, balloon angioplasty is the current treatment of choice. The results of revascularization are quick, and blood pressure control improves rapidly [2,4].

## Case presentation

An African American female in her 60s presented with sudden-onset sharp abdominal pain in the right upper and lower quadrants. The patient had a history of hypertension, which was recalcitrant to medical therapy with three oral antihypertensives (amlodipine, enalapril, and hydralazine), with home blood pressure measurements consistently around 200 mmHg (systolic). There was no known history of diabetes mellitus or atherosclerosis. She was an active smoker, predominantly smoking marijuana. Surgical history was non-contributory. On presentation, her systolic blood pressure was >200 mmHg. In addition to her home medications, she was treated with intravenous labetalol and hydralazine with little effect. Erythrocyte sedimentation rate (ESR), C-reactive protein (CRP), and lactate dehydrogenase (LDH) levels were found to be elevated, with all other hematological and biochemical tests being normal. The estimated glomerular filtration rate (eGFR) was noted to be normal (Table 1).

**Table 1:** Laboratory values of note LDH is an enzyme that helps the body convert food into energy. It is also known as lactic acid dehydrogenase. eGFR is a blood test that measures how well your kidneys are functioning. It is a calculation based on your blood creatinine levels, age, sex, and body size. LDH: lactate dehydrogenase, eGFR stands for estimated glomerular filtration rate, ESR: erythrocyte sedimentation rate, CRP: C-reactive protein

Test	Value	Range
ESR (mm/hour)	80	0-20
CRP (mg/dL)	16.6	<0.3
LDH (U/L)	1,126	140-280
eGFR (mL/minute/1.73 m^2^)	>60	>60

**Figure 1:**
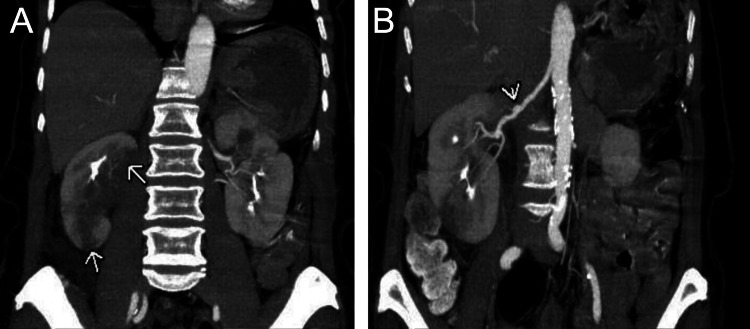
Coronal CTA A: Large wedge-shaped regions of hypoperfusion in the upper pole and lower pole of the right kidney with sharp demarcated margins (arrows). Differential includes renal infarcts, pyelonephritis, and neoplasm. B: Given the beaded appearance of the right renal artery, renal infarct from FMD is likely (arrow). CTA: computed tomography angiography

A CTA was performed (Figure 1), which demonstrated focal aneurysmal dilation of the mid-segment of the right renal artery and two wedge-shaped hypoperfusions of the right kidney, likely secondary to infarcts.

**Figure 2:**
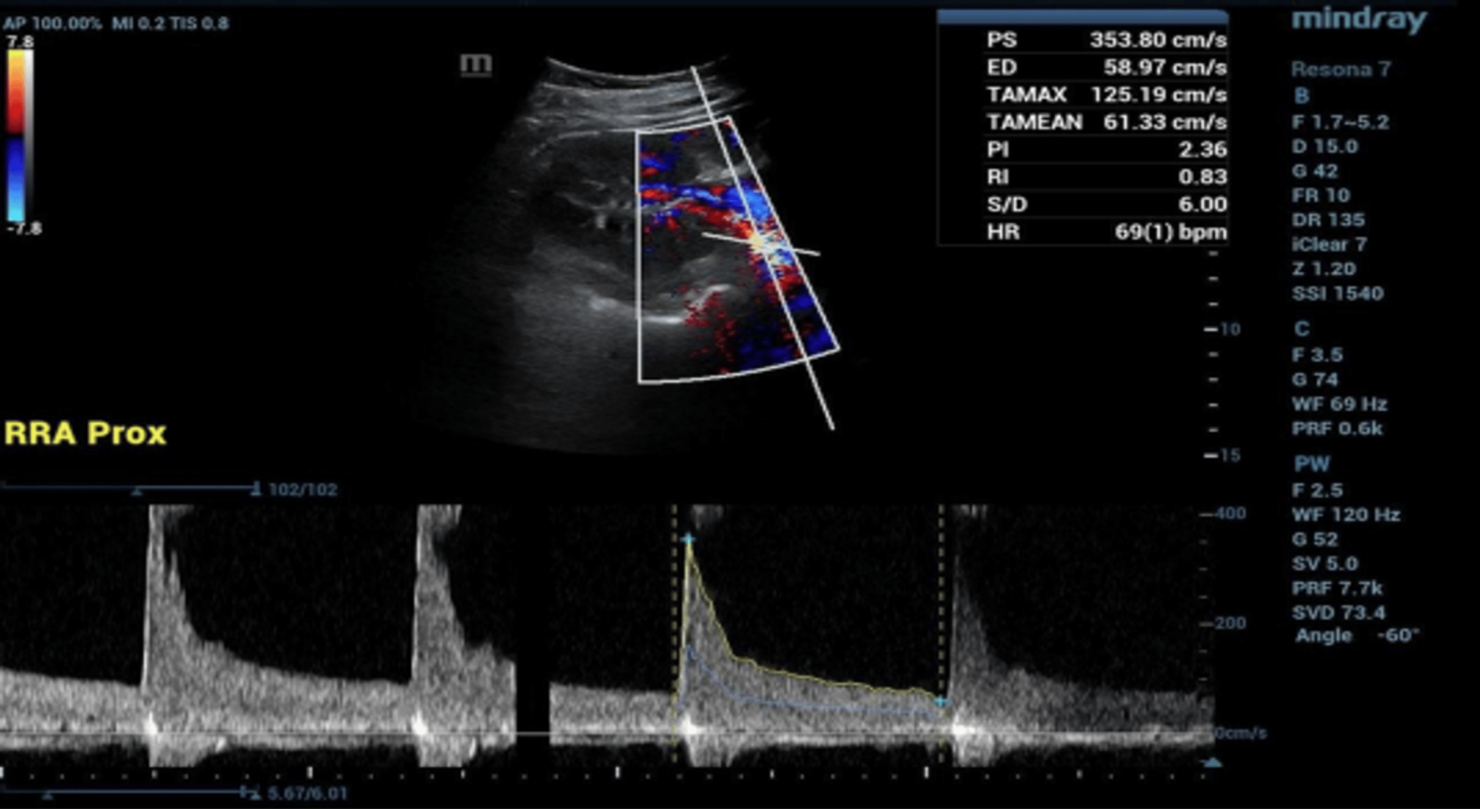
Right renal artery vascular duplex ultrasound Peak systolic velocities were 353.8 cm/s proximally, 330 cm/s in the mid-right renal artery, and 291.4 cm/s distally. These measurements suggest stenosis of the right renal artery greater than 60%. The renal resistive index was 0.7. The renal-aortic ratio was 3.1.

A renal artery duplex ultrasound (Figure 2) suggested >60% stenosis on the right, although the velocities were elevated on the left, too. On the right side, the peak systolic velocity was 359 cm/s, and the renal-aortic ratio was 3.1. Anticoagulation was administered with intravenous heparin infusion at 14 units/kg/hour.

**Figure 3:**
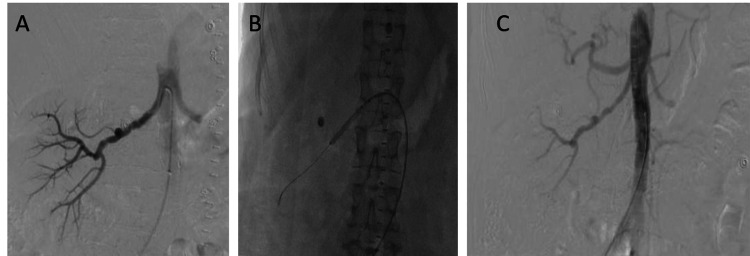
Selective renal angiogram and angioplasty A: Pre-angioplasty image of the right renal artery demonstrating a typical chain of beads appearance, suggestive of fibromuscular dysplasia. B: Balloon angioplasty performed with a 5 × 40 mm balloon. C: Post-angioplasty image showing improvement.

A bilateral selective renal angiogram (Figure 3) was performed, which revealed a classic "string of beads" micro-aneurysmal appearance of the right renal artery but demonstrated a normal appearance of the left renal artery. No thromboemboli were found in the right renal artery; however, areas of hypoperfusion were seen in the upper and lower pole of the right kidney, corresponding with the infarcts seen on CT. Balloon angioplasty was performed with a 5 × 40 mm balloon catheter. Post-procedurally, the patient did well and was discharged home. Approximately two months post-intervention, the patient reported improvement to home blood pressure measurements with most values around 130 mmHg (systolic) with no change in the antihypertensive medication regimen.

## Discussion

FMD is most frequently diagnosed in women aged 15-50 years but can first present in patients after the age of 60, as in this case [4]. Elderly patients with FMD are more likely to be asymptomatic [9], although this was not the case in our patient. The incidence of African Americans with FMD is lower (11.2 per 100,000) than that of Caucasians (15.8 per 100,000), and Caucasians make up 80.5% of patients with FMD [10]. African Americans with FMD are more likely to be male, but there is no difference in renal infarct prevalence between races [10]. This African American female patient presented with FMD in her 60s, demonstrating an uncommon demographic for this disease. In obtaining the patient's social history, it was revealed that she had significant difficulty attending scheduled office appointments secondary to a lack of transportation and chronic musculoskeletal pain exacerbated by travel. When queried, the patient admitted to missing many of her physician appointments due to these factors. In effect, our patient had significantly limited access to healthcare resources, demonstrating a possible explanation for the delayed recognition and workup of FMD. This highlights the role played by social disparities in health outcomes.

This case is a unique presentation of FMD in that the patient presented with renal infarcts. Renal infarcts occur most commonly due to thromboembolism and less commonly with other disorders of coagulation and vessel wall but are rarely seen in FMD [3]. This patient presented with acute, sudden-onset right-sided abdominal pain consistent with pain due to a right renal infarct. The reported annual incidence of abdominal pain due to renal infarcts presenting in emergency departments is 0.007%, while the incidence of renal infarcts in FMD is believed to be 0.9% [10].

Of FMD cases presenting with renal artery involvement, 35%-50% are bilateral, while this case was unilateral FMD of the right renal artery [1-3]. The "string of beads" appearance on this patient's angiogram is consistent with medial fibroplasia, the most common (80%-90%) lesion type in FMD, which affects the middle to distal segments of the renal artery [1,2,4,11]. The dilations in medial fibroplasia are larger than the diameter of the vessel, while in perimedial fibroplasia, focal constrictions are seen [2,9]. Renal infarcts in FMD more commonly occur with perimedial fibroplasia than medial fibroplasia, rendering this case an uncommon presentation [12].

Contrary to the findings in this case, atherosclerosis of the renal artery generally involves the origin or proximal segment of the renal artery in older patients with risk factors for cardiovascular disease [1,4]. Acute phase reactants such as erythrocyte sedimentation rate and C-reactive proteins are expected to be within normal limits in FMD as opposed to vasculitis [4], but the presence of a renal infarct in this patient complicates this determination. This patient's clinical findings and imaging are consistent with a diagnosis of FMD affecting the unilateral renal artery.

As in this case, clinical presentation with non-invasive imaging (e.g., CTA and ultrasonography) allows the diagnosis of FMD with balloon angioplasty remaining the standard first-line treatment [2,4]. The results of balloon angioplasty may be subtle on re-imaging [2], as in our case. We did not have the facilities to measure pressure gradients, but the clinical outcome suggests that the angioplasty was successful.

## Conclusions

We discuss a rare presentation of unilateral fibromuscular dysplasia of the right renal artery complicated by a renal infarct. Renal infarcts are rarely seen in FMD, with only a handful of known cases to date, and usually occur in perimedial fibroplasia, which was not consistent with the findings in this patient. Only about half of the cases of FMD that affect the renal arteries are unilateral. Finally, our patient was older than the typical age range for initial presentation; this could possibly be due to reduced access to healthcare. In combination, these factors lead to a particularly unique presentation of FMD. Renal infarcts, while rare, can be a presenting symptom of FMD. While most patients are diagnosed at a young age, limited access to medical care can preclude workup until late in the disease course. FMD should be considered in the differential diagnosis of refractory hypertension, irrespective of age and race.
